# CD8 T Cell Epitope Distribution in Viruses Reveals Patterns of Protein Biosynthesis

**DOI:** 10.1371/journal.pone.0043674

**Published:** 2012-08-27

**Authors:** Carmen M. Diez-Rivero, Pedro A. Reche

**Affiliations:** Laboratory of Immunomedicine, Department of Immunology, Facultad de Medicina, Universidad Complutense de Madrid, Madrid, Spain; University of Sydney, Australia

## Abstract

Distinguishing T cell epitope distribution patterns is relevant for epitope-vaccine design. To that end, we invest0069gated the distribution of known CD8 T cell epitopes from Hepatitis C Virus, Human Immunodeficiency Virus-1 and Influenza A Virus using χ^2^ statistics. We found that epitopes are not distributed in the viral proteomes proportionally to the size of the source proteins. Specifically, capsid and matrix proteins pack significantly more epitopes than those expected by their size. Such non-homogeneous distribution cannot be accounted by underlying MHC I-peptide binding preferences nor it is related to sequence variability. Instead, we propose that it might be related to preferential protein translation/biosynthesis. Overall, these results support the prioritization of structural antigens for epitope identification and vaccine design.

## Introduction

CD8 cytotoxic T cells play a key role in the defense against intracellular pathogens and tumor cells. CD8 T cell immune responses are driven by the recognition of foreign peptides presented by major histocompatibility complex class I (MHC I) molecules at the cell surface [1]. The identification of these peptides (CD8 T cell epitopes) is therefore important for understanding disease pathogenesis and etiology as well as for vaccine design.

Purely experimental identification of T cell epitopes is costly and time consuming: it requires the synthesis of overlapping peptides spanning the entire length of the protein, followed by complicated *in vitro* cellular assays on each synthesized peptide [2]. Therefore, we, and others, have developed computational approaches to predict T cell epitopes that reduce the experimental load involved in epitope identification. The main basis for anticipating CD8 T cell epitopes is the prediction of MHC I-binding peptides [3]. This approach can also be combined with methods that model other relevant step of the MHC class I antigen processing pathway, such as cleavage by the proteasome [4] and TAP mediated transport [5]. Such combination can improve the epitope predictions obtained considering just peptide binding to MHC I [6]. However, epitope prediction tools are yet far from perfect and generally only 10% of the predicted epitopes are immunogenic (able to elicit a T-cell response) [7], [8]. Therefore, in order to accelerate epitope identification and translational vaccine research, we must improve epitope prediction methods. Additionally, it is key to define rationales for prioritizing protein antigens for epitope prediction and vaccine design [9]. To that end, we analyzed the distribution of known CD8 T cell epitopes.

We focused on three viruses of great clinical relevance: Hepatitis C Virus (HCV), Human Immunodeficiency Virus-1 (HIV) and Influenza A Virus (IAV). Briefly, HCV is a member of the flaviviridae family, which often produces a chronic infection that can lead to cirrhosis and hepatocellular carcinoma. It has a small RNA genome encoding a single polyprotein that is processed into 10 proteins [10], consisting of three structural proteins (core or nucleocapsid, E1 and E2) and seven nonstructural proteins (NS1, NS2, NS3, NS4a, NS4b, NS5a and NS5b). HIV-1 (hereafter HIV) is a lentivirus that causes acquired immunodeficiency syndrome (AIDS) [11]. HIV is composed of two copies of single-stranded RNA, encompassing 9 gene products (Gag, Pol, Vif, Vpr, Tat, Rev, Vpu, Env and Nef), each of producing one of more viral proteins after processing. For example, p17 (MA, matrix protein), p24 (CA, capsid protein), p7 (nucleocapsid protein) and p6 are all produced after the Gag polyprotein. Finally, IAV is a member of the Orthomyxoviridae family with eight single (non-paired) RNA strands encoding of a total of eleven proteins (PB2, PB1, PB1-F2, PA, HA, NP, NA, M1, M2, NS1 and NS2) [12] Each RNA encodes one or more protein products. For example, the RNA segment 7 encodes M1, the matrix protein that forms the viral envelope, and M2, an integral membrane protein. Using reference strains of these three viruses, we mapped and analyzed the location of the HCV-, HIV- and IAV–specific CD8 T cell epitopes onto the viral proteomes, concluding that CD8 T cell epitopes are not evenly distributed. Notoriously, we found that structural proteins Core (HCV), Gag (HIV) and M1 (IAV) pack significantly more peptides than those expected by their size. Here, we will interpret and discuss the significance of these results.

## Results

### Distribution of CD8 T Cell Epitopes

**Figure 1:**
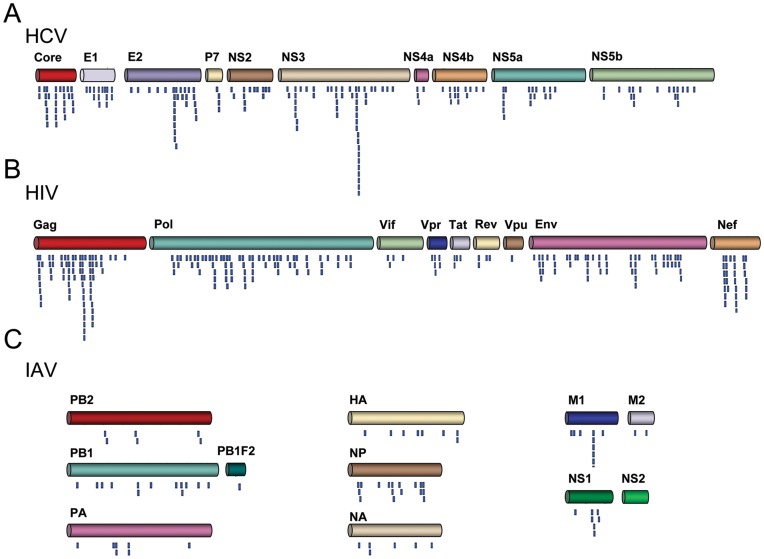
Epitope map. The figure shows the localization of known CD8 T cell epitopes specific of HCV (Panel **A**), HIV (Panel **B**) and IAV (Panel **C**). Epitopes are shown as blue segments underneath of the relevant proteins. IAV proteins that are encoded by the same RNA segment are shown in near proximity. CD8 T cell epitopes used in this work range from 9 to 10 residues and they all differ in at least one amino acid residue (See Material and Methods for details). Therefore, those epitopes that match in the same or near the same location are either epitope variants or overlapping epitopes.

T cell epitopes are small peptide fragments obeying to rules for processing and MHC presentation that are not conceived to be highly specific. Hence, the bigger the protein the larger the number of epitopes that one can expect. Here, we used a χ^2^ test to examine whether CD8 T cell epitopes specific of HCV, HIV and IAV follow a homogeneous protein-size wise distribution. We proceeded as follows. We first mapped the collected epitopes of HCV (190), VIH-1 (249) and IAV (78) onto their relevant proteins (Figure 1), tallying up the number of epitopes that falls within each viral protein (observed epitopes)(Table 1). Next, we distributed the total number of observed epitopes, into the viral proteins proportionally to their length/size, thus getting the number of expected epitopes (Table 1).

**Table 1:** Protein-size distribution of CD8 T cell epitopes in HCV, HIV and IAV.

HCV					
Protein	Protein length	*CF* *	Observed epitopes	Expected epitopes	?^2^
Core	191	0,95	28	11.99	21.36
E1	192	0,58	14	12.12	0.29
E2	364	0,71	27	22.91	0.73
p7	64	0,68	4	3.98	0.001
NS2	218	0,59	13	13.70	0.04
NS3	632	0,89	50	39.83	2.59
NS4a	55	0,83	4	3.41	0.10
NS4b	262	0,84	14	16.47	0.37
NS5a	449	0,75	17	28.28	4.50
NS5b	592	0,81	19	37.31	8.98
Total	3019		190	190	38.97
**HIV**					
**Protein**	**Protein length**	***CF*** *	**Observed epitopes**	**Expected epitopes**	**χ^2^**
Gag	500	0,68	75	39.73	31.32
Pol	1001	0,84	72	79.53	0.07
Vif	192	0,75	4	15.25	8.30
Vpr	96	0,74	6	7.63	0.35
Tat	86	0,63	4	6.75	1.12
Rev	116	0,57	4	9.22	2.95
Vpu	82	0,45	1	6.51	4.67
Env	856	0,54	55	68.01	2.49
Nef	206	0,62	28	16.37	8.27
Total	3135		249	249	60.19
**IAV**					
**Protein**	**Protein length**	***CF*** *	**Observed epitopes**	**Expected epitopes**	**χ^2^**
PB2	759	0,98	6	13.05	3.81
PB1	757	0,1	13	13.01	0.00
PB1F2	87	0,84	1	1.49	0.16
PA	716	0,98	7	12.31	2.29
HA	566	0,88	8	9.73	0.31
NP	498	0,99	17	8.56	8.32
NA	452	0,92	6	7.81	0.42
M1	252	0,99	11	4.33	10.26
M2	97	0,89	2	1.67	0.07
NS1	230	0,83	7	3.95	2.35
NS2	121	0,92	0	2.08	2.08
Total	4537	10,22	78	78	30.06

The expected epitopes in a given protein are those resulting after distributing all of the virus-specific epitopes proportionally to the length of that protein with regard to the total size of the relevant viral proteome.

* Conservation Factor of each protein.

**Figure 2:**
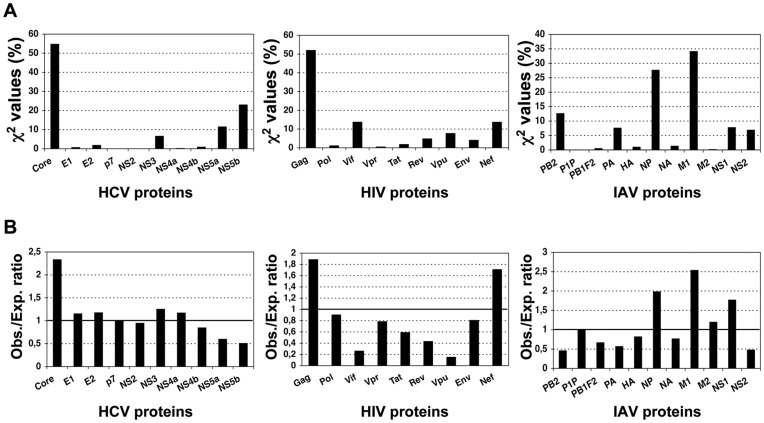
Protein-size distribution of virus-specific CD8 T cell epitopes. We depict for each of the viral proteins of HCV, HIV and IAV the contribution (in percentage) to the χ^2^ statistics (Panel **A**) and the ratio between observed and expected epitopes (Panel **B**). A value greater than 1 indicates more observed epitopes than expected, while a value lower than 1 reflects fewer epitopes than expected.

The results of the χ^2^ test showed that the distribution of the CD8 T cell epitopes is not proportional to the size/length of the proteins (α = 0.001) in any of the viral proteomes studied here (HCV: χ^2^_9,0.001_ = 27.88<χ^2^ 38.97, *p* = 1.66 10^−5^; HIV: χ^2^_8,0.001_ = 26.12<χ^2^ = 60.19, *p* = 4.27 10^−10^; IAV: χ^2^_10,0.001_ = 29.59<χ^2^ = 30.06, *p* = 0.00084). To better visualize such uneven distribution, we represented the contribution of each protein, in percentage, to the χ^2^-statistic value (Figure 2A), and the ratio between observed and expected epitopes in each protein (Figure 2B). In Figure 2B, a ratio >1 indicates more observed epitopes than expected, whereas a ratio <1 indicates the opposite (fewer epitopes than expected). The most significant differences were found in non-enzymatic structural proteins of the viruses; their contribution to the χ^2^ statistics is nearly enough to reject the null hypothesis (Figure 2A). These proteins carry more epitopes than the expected by their size. Thus, HCV Core protein encompasses 2.3-fold more epitopes than expected (Figure 1B) and Gag protein, which includes several non-enzymatic HIV-1 structural proteins, has 1.9-times more epitopes than expected (Figure 2B). Finally, the matrix M1 protein of IAV also encompasses 2.5-times more epitopes than the expected by their size (Figure 1B). Likewise, NP encompasses 2-times more epitopes than expected (Figure 2B).

Other proteins also contributed significantly to the χ^2^-statistics (Figure 2A). In HCV, NS5a and NS5b bear 1.6- and 1.9-times, respectively, fewer epitopes than expected (Figure 2B). In HIV, Vif and Rev encompass 3.8-times and 2.3-times fewer epitopes than expected (Figure 2B). An interesting case to comment is that of HIV-1 Vpu protein. As shown in Figure 1B, Vpu exhibits 6.5-times fewer epitopes than expected, the largest difference observed. Nonetheless, this difference does not have a major contribution to the χ^2^-statistics (Figure 2A) as Vpu only bears a minor proportion of all HIV epitopes.

**Figure 3:**
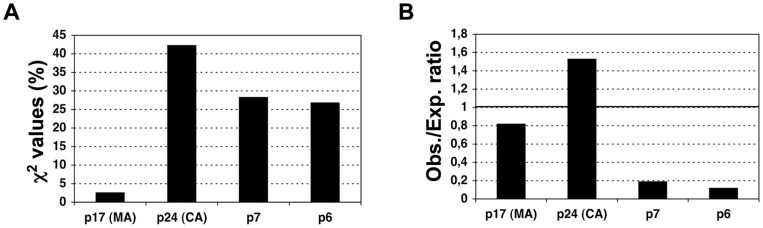
Protein-size distribution of Gag-specific CD8 T cell epitopes. In panel **A**, we show the contribution of p17, p24, p7 and p6 to the Gag χ^2^ statistics and in panel **B**, the ratio between observed and expected epitopes.

In HCV and IVA, the structure-building proteins Core and M1, respectively, that pack more epitopes than the expected by their size are present in the mature viruses. In IAV, M1 is translated after one of the two alternative mRNAs that are produced after the M RNA segment 7 [12]. In HCV, Core is located at the beginning of a single translated open reading frame (ORF). In HIV-1, the Gag protein, in which we also found more epitopes than expected, is actually processed during maturation to produce four different viral proteins: p17 (MA, matrix), p24 (CA, capsid), p7 and p6 (from the N-terminus to the C-terminus). Therefore, we also used the described χ^2^-test to analyze the distribution of the 75 Gag-specific CD8 T-cell epitopes within the relevant proteins at same α-value than before (0.001). The results clearly show that Gag-specific epitopes are not distributed proportionally to protein size/length (χ^2^_3,0.001_ = 16.27<χ^2^ = 20.31, *p* = 0.0001). The most relevant contributions to the χ^2^-statistics are observed in protein p24 (CA) and p6 (Figure 3A). Protein p24 encompasses 1.5-times more epitopes than the expected while p6 bears 8.4-times fewer epitopes than expected (Figure 3B).

### Distribution of MHC I Binding Sites

**Figure 4:**
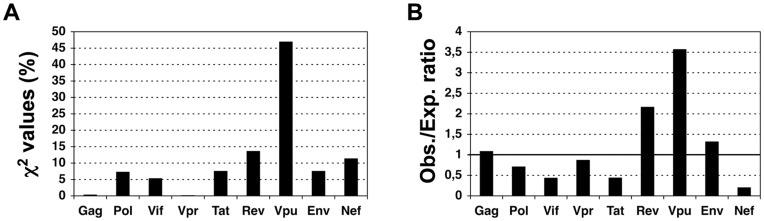
Distribution of predicted A*0201-binding peptides in HIV. Only A*0201-bindig peptides from HIV were not distributed homogeneously by protein size at the α value (0.001) used in the epitope analysis. In panel **A**, we show the contribution (in percentage) to the χ^2^ statistics of each HIV protein and in panel **B**, the ratio between observed and expected A*0201-binding peptides.

We wished to examine whether the noted non-homogeneous distribution of T cell epitopes in the viral proteomes mirrored underlying MHCI binding preferences. To that end, we targeted for peptide binding predictions three human MHCI molecules, HLA-A*0201, HLA-A*0301 and HLA-B*0702 (details in Materials and Methods). A*0201, A*0301, B*0702 belong to the A2, A3 and B7 HLA I supertypes, respectively. These HLA I supertypes are expressed in about 90% of population and have peptide binding repertoires that are largely non-overlapping [13]. Then, we used the χ^2^ test, as described earlier, to analyze the distribution of peptides predicted to bind to A*0201, A*0301 and B*0702, individually to each MHC I molecules and in combination. In Table S1, we provide the detailed analysis. Unlike CD8 T cell epitopes, we found that the predicted MHCI-binding peptides are largely distributed proportionally to the length of the proteins (Table 2). This result is the expected: the larger the protein the larger the number of potential peptide binders to MHC I. In fact, at an α-value of 0.001 (that used in the CD8 T cell epitope analysis), only A*0201 binding peptides in HIV are not distributed homogeneously with regard to protein size (χ^2^_8,0.001_ = 26.12<χ^2^ = 27.59, *p* = 0.0006). However, the distribution of HIV-specific A*0201 binding peptides (Figure 4) does not match the epitope distribution (Figure 2). For instances, the major contribution to the non-homogeneous distribution of the A*0201-binding peptides lies in Vpu which encompasses 3.6-fold more binding peptides than expected (Figure 4B), whereas Vpu carries fewer epitopes than expected (Figure 2B). Moreover, the most important contribution to the non-homogenous distribution of the observed epitopes lies in Gag, in which the number of A*0201-binding peptides does not differ from the expected. At a more permissive α of 0.01, we find that peptides binding to A*0201 in HCV are neither distributed proportionally to the length of the proteins (χ^2^_9,0.01_ = 21.67<χ^2^ = 22.56, p = 0.0073). In this case, the most notorious influence to the statistic is seen in NS4a, in which the number of predicted A*0201-binding peptides exceed the number of expected binders (Table S1), again the opposite to that seen with the epitopes (Figure 2B). The combination of the peptides predicted to bind to A*0201, A*0301, B*0702 always followed a homogenous distribution proportional to the size of the source proteins (*p*>0.05, Table 2).

**Table 2:** χ^2^–statistics resulting of analyzing the distribution of MHC I-binding peptides in HCV, HIV and IAV.

	HCV		HIV		IAV	
MHC I-bindingpeptides to:	?^2^	*p-value*	?^2^	*p-value*	?^2^	*p-value*
A*0201	22.56	0.0073	27.59	0.0006	18.1	0.053
A*0301	16.96	0.049	4.45	0.81	20.12	0.0281
B*0702	16.48	0.058	2.48	0.96	12.2	0.27
A*0201+ A*0301+ B*0702*	11.2	0.26	13.4	0.096	11.15	0.346

* Statistics obtained with sum of the peptides that are predicted to bind each MHC I molecule.

### Epitope Distribution and Sequence Conservation

**Figure 5:**
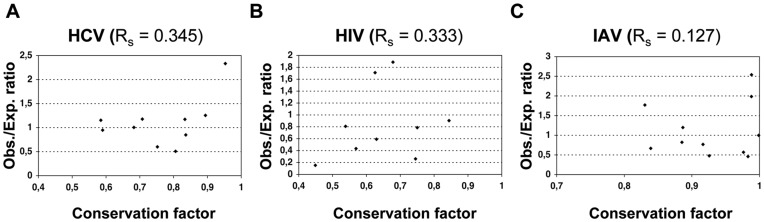
Correlation between epitope distribution and sequence conservation. For the proteomes of HCV (panel **A**), HIV (panel **B**) and IAV (panel **C**), we plot the ratio between observed and expected epitopes (Y-axis) against the corresponding conservation factors (*CF*)(X-axis).

Variable proteins likely bear multiple epitope variants that have not been identified. As result, the epitope distribution that we can obtain using a set of known CD8 T cell epitopes may be conditioned by protein sequence variability. Therefore, we examined the correlation between sequence conservation and epitope distribution. To that end, we computed a protein conservation factor (CF) (details in Materials and Methods) for each of the viral proteins and studied their correlation with the corresponding ratio between observed and expected epitopes, using Spearman’s rank correlation (*R_s_*) (Figure 5). The largest correlation was found in HCV (*R_s_* = 0.345), followed by HIV (*R_s_*_ = _0.333) and IAV (*R_s_* = 0.127). However, all of the correlation values were very small and in fact none of the then was statistically different from zero.

## Discussion

Distinguishing T cell epitope distribution patterns is relevant for epitope-vaccine design. However, to the best of our knowledge, there is little or no evidence on whether T cell epitopes are distributed in any preferential manner onto pathogens’ proteomes. Therefore, we investigated this matter in three human viruses, HCV, HIV and IAV, encompassing the largest known collections of CD8 T cell epitopes. Mapping of CD8 T cell epitopes onto the relevant viral proteomes did not reveal any obvious pattern and, in general, the larger the proteins the more epitopes they carry (Figure 1). However, using a χ^2^ test we found that CD8 T cell epitopes are not distributed homogeneously proportional to the size of the proteins. Specifically, structural proteins assembling the viral capsid such as Core in HCV and Gag p24 in HIV display more epitopes than the expected for their size (Figure 2 and Figure 3). Likewise, matrix proteins including M1 of IAV also bear more epitopes than expected (Figure 2). At the other end, there are viral proteins such as NS5a and NS5b in HCV, Vif and Vpu in HIV and PB2 in IAV that display fewer epitopes than the expected by their size (Figure 2). T cell epitopes consist of peptides that need to bind and be presented by MHC I molecules prior to T cell recognition. However, in contrast to the analyzed epitopes, we found that MHC I-binding peptides are largely distributed proportionally to the size of the source of viral proteins (Table 2) and does not mirror the distribution pattern of epitopes (Figure 4). Therefore, the observed epitope distribution does not appear to obey to any underlying MHCI binding preferences.

Another factor that can shape epitope distribution patterns is sequence variability. Experimental verification of epitopes (as those used here) requires determining T cell responses against synthetic peptides and responses elicited against variant epitopes will pass undetected [14]. Therefore, there could be a bias in known CD8 T cell epitopes towards conservation that could lead to observe fewer epitopes than expected in variable proteins and more than expected in conserved proteins. However, we did not find any significant correlation between the epitope distributions described here and sequence conservation (Figure 5). Therefore, sequence conservation/variability does not explain the noted epitope distribution. Arguably, HLA I bias in the datasets may also affect the noted epitope distribution. In fact, A*0201-restricted epitopes are overrepresented in our datasets (See Materials and Methods). However, if we discard all A*0201-restricted peptides from our datasets the epitope distributions remain largely the same (Table S2). Although we cannot discard that our results might reflect bias of researchers towards studying specific viral proteins, it appears that epitope skewing relates to protein expression: structural proteins from virus are expressed at high levels. Moreover, we find worth noting the following observation. In HCV and HIV, the proteins that bear more epitopes than expected (structural proteins) are located near or at the N-terminus of protein products encompassing other viral proteins that get translated together from a single open reading frame (ORF). Conversely, those proteins located at the C-terminus bear fewer epitopes than expected (Figure 2 and Figure 3). The extreme paradigm is HCV, whose entire proteome is made upon a single polyprotein, which is translated from a single ORF. In this polyprotein, the structural protein Core is located at the N-terminus and N5Sb at the C-terminus. We can make some interesting inferences from this observation.

Peptides presented by MHC I molecules –and thereby CD8 T cell epitopes – are derived from the degradation of newly synthesized defective ribosomal products (DRiPs) and degradation of mature proteins as part of their turnover [15], [16]. However, in general, the major portion of peptides presented by MHC I molecules derives from DRiPs, which are quickly degraded [16], [17], [18], [19], [20]. Thus, protein translation plays a major role in the generation of peptides for presentation by MHC I: protein translation is a very inefficient process and the more translation the more peptides available for presentation. Therefore, that we find more epitopes than expected in proteins located near the beginning of translated ORFs (*e.g.* Core in HCV), and fewer in those located at the end (*e.g.* N5Sb), suggest that the ribosome must often fail to translate full ORFs, resulting in incomplete protein products. As a result, proteins located at the beginning of ORF get synthesized/translated predominantly, thus, providing a major source of peptides for antigen presentation. Conversely, if ribosomes would consistently synthesize the full polyprotein, we should have instead found more epitopes than expected in viral proteins expressed in low copy numbers that are found at the C-terminus of the polyprotein, as they would have been subjected to a greater degree of degradation (*e.g.* N5Sb).

The simplicity of viruses calls for simple and yet effective mechanisms of protein expression regulation. Thus, placing the structural proteins at the beginning of translated ORF, such as the case of HCV, is likely a means to guarantee the high copy numbers required for the assembly of the virus. To our knowledge, this simple position-based translational control of protein expression levels has not been described before and it will require experimental confirmation. A similar mechanism but acting at the transcriptional level has been described in negative-strand RNA viruses. In these viruses, levels of gene expression are primarily regulated by the position of each gene relative to the single promoter and also by cis-acting sequences located at the beginning and end of each gene and at the intergenic junctions [21]. One could argue that protein stability and turnover could also provide an alternative mechanism to explain protein expression levels. Under this scenario, structural proteins would be present at high copy numbers simply because they are very stable and have a low turnover rate. In fact, structural proteins are most likely very stable and such stability can surely contribute to keep their expression levels high. However, the epitope distribution supporting that protein expression levels in HCV are controlled by protein stability and/or turnover would be just the opposite to that observed. Namely, there should be fewer epitopes than expected in HCV proteins that are expressed at high copy numbers (*e.g.* Core protein) and more epitopes than expected in those that are expressed at low copy numbers (*e.g.* N5Sb).

That CD8 T cell epitopes are preferentially located in viral structural proteins, which, incidentally, are generally expressed in high numbers and are often conserved and translated in first place, has profound implications for vaccine development against viruses. In fact, it supports that structure building protein antigens ought to be prioritized for T cell epitope prediction/identification and vaccine development. Such antigen prioritization ought to save time and resources needed for epitope-vaccine development. It is important to remark that in this study we have not considered the level of immunogenicity of the epitopes. Our datasets included inmunodominant (more immunogenic) and subdominant (less immunogenic) epitopes. It would have been interesting to investigate whether epitope immunogenicity condition their distribution. However, epitope immunogenicity relativeness and lack of relevant data precluded that effort. Epitope immunogenicity is conditioned by many factors including other HLA molecules and previous pathogenic encounters [22]. Thus, two identical individuals do not need to respond to the same epitopes and the targeted epitopes often change through the course of an infection [23]. On the other hand, as vaccine design is concerned, one should not disregard the relevance of subdominant epitopes as immunodominance can be reverted through vaccination [24], [25] and the most immunogenic epitopes are not necessarily those providing protection. In fact, the epitopes that can elicit a protective CD8 T cell response are those that can be processed from their source antigens and be presented by the relevant MHC I molecule, in both, the antigen presenting cells priming the CD8 T cells and their target cells hosting the infection [7], [26]. Therefore, for designing protective vaccines that incorporate CD8 T cell epitopes, rather than identifying/using immunodominant epitopes one needs to focus on identifying epitopes that meet the following criteria: conservation, when sequence variability is a strategy for immune evasion, and shared processing and presentation by the antigen presenting cells and the target cells. Naturally, epitope-based vaccines will also need to incorporate CD4 T cell epitopes, which, following this analysis, should also be identified from the same structure building proteins.

The present rational for antigen prioritization has been drawn from the T cell epitope distribution in three viruses and it is meant for viruses. However, CD8 T cells play also a role in conferring protection against some intracellular bacteria (*e.g. Listeria*) and some protozoan parasites (*e.g. Plasmodium*) [27], [28] and it is reasonable to wonder whether the same –or similar– rational can be applied to these pathogens. Namely, whether those antigens that are expressed at high levels should be prioritized for epitope identification and vaccine design. That could well be the case but its confirmation will require further investigation as it not strictly supported by the present analysis: both, bacteria and protozoan parasites, have their own protein translation (ribosomes) and degradation machinery (*e.g.* proteasomes), and such machinery is not involved, at least directly, in providing peptides for presentation by MHC I.

## Materials and Methods

### CD8 T Cell Epitope Sets

We used three sets of CD8 T cell epitopes specific of HIV, HCV and IAV, encompassing 190, 249 and 78 epitopes, respectively. The datasets consisted of unique peptides of 9 or 10 residues that were collected from EPIMHC [29], Immuneepitope [30] and Los Alamos HIV databases (www.hiv.lanl.gov/). We only selected epitopes that were reported to be restricted by human MHC I molecules and are able to elicit immune responses in the course of a natural infection in humans. These datasets are now provided in File S1.

### Reference Sequences and Epitope Mapping

We applied a fuzzy pattern-matching algorithm based on the *String::Aprox* - Perl extension, allowing a maximum of 3 substitutions, for mapping CD8 T epitopes in representative reference amino acid sequences of the viral proteins of HCV, HIV, IAV. Reference sequences were obtained from the following GenBank accession number: NC_009827.1 for HCV (genotype 6), NC_001802.1 for HIV-1 strain HXB2. For IAV, we used the sequences given by the accessions NC_004905 to NC_004912, specific for the 8-genomic segments of the Hong Kong/1073/99(H9N2) strain.

### Protein Conservation Factor

We computed a protein conservation factor (*CF*) for each of the proteins encoded by HCV, HIV and IAV using equation 1:
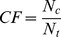
(1)where *N_c_* is the number of non-variable residues and *N_t_* the total number of amino acids of the protein. *CF* ranges between 0 and 1, taking the value of 1 when the protein has no variable residues. Non-variable residues were identified from the relevant protein sequence alignments as those with a Shannon entropy (*H*) ≤1 [31], [32]. Shannon entropy per site was computed using equation 2.
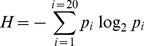
(2)where Pi is the fraction of residues of amino acid type i. H ranges from 0 (total conservation, only one amino-acid type is present at that position) to 4.322 (all 20 amino acids are equally represented in that position).

Multiple sequence alignments (MSAs) required for computing sequence variability were obtained as follows. For the IAV, we used the reference genome NC_002016–NC_002023 and BLAST each of the encoded proteins against a BLAST database built upon IAV proteins from strains H5, H7 and H9 obtained from NCBI. Subsequently, we realigned the sequences resulting of the BLAST searches using TCOFFEE [33]. For HCV and HIV-1, we retrieved the relevant alignments from Los Alamos HIV database and realigned them using TCOFFEE.

### Statistical Analyses

We used χ^2^ goodness of fit test to assess whether the distribution of the epitopes in the proteins of HCV, HIV and IAV was uniform–proportional to the size of the proteins– or not. The χ^2^-statistic value was computed by equation 3.
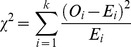
(3)where *k* is the number of proteins, *O_i_* is the number of observed epitopes in protein *i*, and *E_i_* is the number of expected epitopes in the protein *i* as if they were distributed proportionally to the size of the proteins. The H_o_ hypothesis (epitopes are distributed proportionally to the size of the proteins) is rejected if the computed χ^2^ statistics exceeds the χ^2^–distribution value at *k –1* degrees of freedom and a given *α* value (χ^2^ *_k−1,_*_α_)

We used permutation tests to assess whether Spearman’s rank correlation coefficients (*R_s_*), obtained upon correlating protein sequence conservation and epitope distribution, were significantly different from zero.

### Prediction of Peptide-MHCI Binding

We used position Specific Scoring Matrices (PSSMs) [34], also known as profiles, to predict peptide binding to the human MHC I molecules HLA-A*0201, HLA-A*0301, HLA-B*0702. We only considered peptide binders of 9 residues in length (9mers). We applied PSSMs to the entire viral proteomes –upon combining all the viral proteins–, comparing the binding score of each peptide to those of 10000 reference peptides (9-mers randomly obtained from SwissProt). A given peptide was considered to bind to an MHC I molecule when its binding score was within the top 2% binding scores.

## Supporting Information

Table S1 Protein-size distribution of MHC I-binding peptides from HCV, HIV and IAV. Predicted MHC I-binding peptides were obtained using the relevant motif profiles, as indicated in Materials and Methods. The expected peptide binders in a given protein are those resulting after distributing all of the relevant binders proportionally to the length of that protein with regard to the total length of the viral proteome. The distribution of MHC I-binding peptides in HCV, HIV and IAV is considered non-homogeneous according to the length of the proteins when the χ^2^ statistic is greater than 27.88, 26.12 and 29.59, respectively, with α = 0.001.
(DOC)

Table S2 Protein-size distribution of CD8 T cell epitopes, excluding those restricted by A*0201, in HCV, HIV and IAV. This table was prepared as Table 1 but the data was obtained after excluding all A*0201-restricted peptides from the CD8 T cell epitope sets. The expected epitopes in a given protein are those resulting after distributing all of the virus-specific epitopes proportionally to the length of that protein with regard to the total size of the relevant viral proteome. CD8 T cell epitope distribution in HCV, HIV and IAV is considered non-homogeneous according to the length of the proteins when the χ^2^ statistic is greater than 27.88, 26.12 and 29.59, respectively, with α = 0.001. * Conservation Factor of each protein.
(DOC)

File S1 CD8 T cell epitope sets. The file shows the amino acid sequence of the CD8 T cell epitopes from HIV, HCV and IAV used in this study. The epitopes were collected from EPIMHC [29], Immuneepitope [30] and Los Alamos HIV databases (www.hiv.lanl.gov/). All epitopes have between 9 to 10 residues, are restricted by human MHC I molecules and were reported to be elicited in the course of a natural infection in humans.
(XLS)
