# Supplementary material for: CD8 T Cell Epitope Distribution in Viruses Reveals Patterns of Protein Biosynthesis
Source: PLoS One. 2012 Aug 27;7(8):e43674. doi: 10.1371/journal.pone.0043674 (PMC3428354; doi:10.1371/journal.pone.0043674)
Supplement: Table S1 — Protein-size distribution of MHC I-binding peptides from HCV, HIV and IAV. Predicted MHC I-binding peptides were obtained using the relevant motif profiles, as indicated in Materials and Methods. The expected peptide binders in a given protein are those resulting after distributing all of the relevant binders proportionally to the length of that protein with regard to the total length of the viral proteome. The distribution of MHC I-binding peptides in HCV, HIV and IAV is considered non-homogeneous according to the length of the proteins when the χ2 statistic is greater than 27.88, 26.12 and 29.59, respectively, with α = 0.001. (DOC) [file pone.0043674.s001.doc]

**Table S1. Protein-size distribution of MHC I-binding peptides in HCV, HIV and IAV**

| **HCV** | | |  |  |
| --- | --- | --- | --- | --- |
| **A*0201-binding peptides** | | |  |  |
| Protein | Protein length | Predicted peptide binders | Expected peptide binders | χ^2^ |
| Core | 191 | 4 | 5.24 | 0.29 |
| E1 | 192 | 6 | 5.29 | 0.09 |
| E2 | 364 | 10 | 10.01 | 0 |
| p7 | 64 | 3 | 1.74 | 0.92 |
| NS2 | 218 | 11 | 5.98 | 4.2 |
| NS3 | 632 | 10 | 13.4 | 3.14 |
| NS4a | 55 | 5 | 1.49 | 8.27 |
| NS4b | 262 | 12 | 7.78 | 3.2 |
| NS5a | 449 | 7 | 12.35 | 2.32 |
| NS5b | 592 | 15 | 16.29 | 0.1 |
| Total | 3019 | 83 | 83 | 22.56 |
| **A*0301-binding peptides** | | |  |  |
| Protein | Protein length | Predicted peptide binders | Expected peptide binders | χ^2^ |
| Core | 191 | 1 | 2.71 | 1.08 |
| E1 | 192 | 4 | 2.74 | 0.58 |
| E2 | 364 | 0 | 5.18 | 5.19 |
| p7 | 64 | 0 | 0.9 | 0.9 |
| NS2 | 218 | 3 | 3.1 | 0.003 |
| NS3 | 632 | 7 | 9.01 | 0.45 |
| NS4a | 55 | 0 | 0.77 | 0.77 |
| NS4b | 262 | 7 | 3.73 | 2.87 |
| NS5a | 449 | 6 | 6.4 | 0.02 |
| NS5b | 592 | 15 | 8.44 | 5.06 |
| Total | 3019 | 43 | 43 | 16.96 |
| **B*0702-binding peptides** | | |  |  |
| Protein | Protein length | Predicted peptide binders | Expected peptide binders | χ^2^ |
| Core | 191 | 6 | 5.74 | 0.01 |
| E1 | 192 | 4 | 5.8 | 0.56 |
| E2 | 364 | 7 | 10.97 | 1.44 |
| p7 | 64 | 3 | 1.91 | 0.63 |
| NS2 | 218 | 1 | 6.56 | 4.71 |
| NS3 | 632 | 21 | 19.08 | 0.19 |
| NS4a | 55 | 1 | 1.63 | 0.24 |
| NS4b | 262 | 9 | 7.89 | 0.15 |
| NS5a | 449 | 24 | 13.54 | 8.07 |
| NS5b | 592 | 15 | 17.87 | 0.46 |
| Total | 3019 | 91 | 91 | 16.48 |
| **Combination: A*0201-, A*0301- and B*0702-binding peptides** | | | | |
| Protein | Protein length | Predicted peptide binders | Expected peptide binders | χ^2^ |
| Core | 191 | 11 | 13.51 | 0.46 |
| E1 | 192 | 14 | 13.65 | 0.01 |
| E2 | 364 | 17 | 25.81 | 3.01 |
| p7 | 64 | 6 | 4.48 | 0.52 |
| NS2 | 218 | 15 | 15.43 | 0.01 |
| NS3 | 632 | 37 | 44.86 | 1.38 |
| NS4a | 55 | 6 | 3.84 | 1.22 |
| NS4b | 262 | 27 | 18.55 | 3.84 |
| NS5a | 449 | 36 | 31.85 | 0.54 |
| NS5b | 592 | 45 | 42.02 | 0.21 |
| Total | 3019 | 214 | 241 | 11.19 |

| **HIV** | | |  |  |
| --- | --- | --- | --- | --- |
| **A*0201-binding peptides** | | |  |  |
| Protein | Protein length | Predicted peptide binders | Expected peptide binders | χ^2^ |
| Gag | 500 | 13 | 11.96 | 0.09 |
| Pol | 1001 | 17 | 23.94 | 2.01 |
| Vif | 192 | 2 | 4.59 | 1.46 |
| Vpr | 96 | 2 | 2.29 | 0.04 |
| Tat | 86 | 0 | 2.08 | 2.08 |
| Rev | 116 | 6 | 2.77 | 3.75 |
| Vpu | 82 | 7 | 1.96 | 12.95 |
| Env | 856 | 27 | 20.47 | 2.08 |
| Nef | 206 | 1 | 4.93 | 3.13 |
| Total | 3135 | 75 | 75 | 27.59 |
| **A*0301-binding peptides** | | |  |  |
| Protein | Protein length | Predicted peptide binders | Expected peptide binders | χ^2^ |
| Gag | 500 | 5 | 5.58 | 0.06 |
| Pol | 1001 | 12 | 11.17 | 0.06 |
| Vif | 192 | 3 | 2.14 | 0.34 |
| Vpr | 96 | 0 | 1.07 | 1.07 |
| Tat | 86 | 1 | 0.97 | 0.001 |
| Rev | 116 | 2 | 1.29 | 0.38 |
| Vpu | 82 | 1 | 0.91 | 0.01 |
| Env | 856 | 11 | 9.55 | 0.22 |
| Nef | 206 | 0 | 2.29 | 2.3 |
| Total | 3135 | 35 | 35 | 4.45 |
| **B*0702-binding peptides** | | |  |  |
| Protein | Protein length | Predicted peptide binders | Expected peptide binders | χ^2^ |
| Gag | 500 | 12 | 11 | 0.09 |
| Pol | 1001 | 22 | 22.02 | 0 |
| Vif | 192 | 6 | 4.22 | 0.75 |
| Vpr | 96 | 1 | 2.11 | 0.58 |
| Tat | 86 | 2 | 1.91 | 0.003 |
| Rev | 116 | 1 | 2.55 | 0.94 |
| Vpu | 82 | 2 | 1.8 | 0.02 |
| Env | 856 | 18 | 18.83 | 0.04 |
| Nef | 206 | 5 | 4.53 | 0.04 |
| Total | 3135 | 69 | 69 | 2.48 |
| **Combination: A*0201-, A*0301- and B*0702-binding peptides** | | | | |
| Protein | Protein length | Predicted peptide binders | Expected peptide binders | χ^2^ |
| Gag | 500 | 30 | 28.54 | 0.07 |
| Pol | 1001 | 51 | 57.14 | 0.66 |
| Vif | 192 | 11 | 10.96 | 0 |
| Vpr | 96 | 3 | 5.48 | 1.12 |
| Tat | 86 | 3 | 4.96 | 0.78 |
| Rev | 116 | 9 | 6.21 | 0.85 |
| Vpu | 82 | 10 | 4.68 | 6.04 |
| Env | 856 | 56 | 48.86 | 1.04 |
| Nef | 206 | 6 | 11.76 | 2.82 |
| Total | 3135 | 179 | 179 | 13.39 |
| **IAV** | | |  |  |
| **A*0201-binding peptides** | | |  |  |
| Protein | Protein length | Predicted peptide binders | Expected peptide binders | χ^2^ |
| PB2 | 759 | 14 | 19.4 | 1.5 |
| PB1 | 87 | 0 | 2.22 | 2.22 |
| PB1F2 | 757 | 20 | 19.35 | 0.02 |
| PA | 716 | 19 | 18.3 | 0.03 |
| HA | 566 | 17 | 14.46 | 0.44 |
| NP | 498 | 8 | 12.73 | 1.75 |
| NA | 454 | 8 | 11.6 | 1.12 |
| M1 | 252 | 14 | 6.49 | 8.65 |
| M2 | 97 | 4 | 2.48 | 0.93 |
| NS1 | 230 | 7 | 5.88 | 0.21 |
| NS2 | 121 | 5 | 3.09 | 1.18 |
| Total | 4537 | 116 | 116 | 18.1 |
| **A*0301-binding peptides** | | |  |  |
| Protein | Protein length | Predicted peptide binders | Expected peptide binders | χ^2^ |
| PB2 | 759 | 18 | 14.38 | 0.91 |
| PB1 | 87 | 5 | 1.65 | 6.81 |
| PB1F2 | 757 | 19 | 14.34 | 1.51 |
| PA | 716 | 16 | 13.56 | 0.43 |
| HA | 566 | 9 | 10.72 | 0.28 |
| NP | 498 | 6 | 9.43 | 1.25 |
| NA | 454 | 1 | 8.6 | 6.72 |
| M1 | 252 | 6 | 4.81 | 0.29 |
| M2 | 97 | 0 | 1.84 | 1.83 |
| NS1 | 230 | 4 | 4.36 | 0.03 |
| NS2 | 121 | 2 | 2.29 | 0.04 |
| Total | 4537 | 86 | 86 | 20.12 |
| **B*0702-binding peptides** | | |  |  |
| Protein | Protein length | Predicted peptide binders | Expected peptide binders | χ^2^ |
| PB2 | 759 | 23 | 14.54 | 4.91 |
| PB1 | 87 | 1 | 1.67 | 0.27 |
| PB1F2 | 757 | 16 | 14.51 | 0.15 |
| PA | 716 | 9 | 13.72 | 1.62 |
| HA | 566 | 8 | 10.85 | 0.75 |
| NP | 498 | 7 | 9.54 | 0.68 |
| NA | 454 | 12 | 8.7 | 1.25 |
| M1 | 252 | 4 | 4.87 | 0.15 |
| M2 | 97 | 2 | 1.86 | 0.01 |
| NS1 | 230 | 5 | 4.41 | 0.08 |
| NS2 | 121 | 0 | 2.23 | 2.32 |
| Total | 4537 | 87 | 87 | 12.19 |
| **Combination: A*0201-, A*0301-, B*0702-binding peptides** | | | | |
| Protein | Protein length | Predicted peptide binders | Expected peptide binders | χ^2^ |
| PB2 | 759 | 55 | 47.99 | 1.02 |
| PB1 | 87 | 6 | 5.5 | 0.04 |
| PB1F2 | 757 | 55 | 47.86 | 1.06 |
| PA | 716 | 44 | 45.27 | 0.0 |
| HA | 566 | 33 | 35.79 | 0.21 |
| NP | 498 | 21 | 31.49 | 3.49 |
| NA | 454 | 21 | 28.71 | 2.07 |
| M1 | 252 | 23 | 16.06 | 2.99 |
| M2 | 97 | 6 | 6.13 | 0.003 |
| NS1 | 230 | 16 | 14.54 | 0.15 |
| NS2 | 121 | 7 | 7.65 | 0.05 |
| Total | 4537 | 287 | 287 | 11.15 |
