# Supplementary material for: CD8 T Cell Epitope Distribution in Viruses Reveals Patterns of Protein Biosynthesis
Source: PLoS One. 2012 Aug 27;7(8):e43674. doi: 10.1371/journal.pone.0043674 (PMC3428354; doi:10.1371/journal.pone.0043674)
Supplement: Table S2 — Protein-size distribution of CD8 T cell epitopes, excluding those restricted by A*0201, in HCV, HIV and IAV. This table was prepared as Table 1 but the data was obtained after excluding all A*0201-restricted peptides from the CD8 T cell epitope sets. The expected epitopes in a given protein are those resulting after distributing all of the virus-specific epitopes proportionally to the length of that protein with regard to the total size of the relevant viral proteome. CD8 T cell epitope distribution in HCV, HIV and IAV is considered non-homogeneous according to the length of the proteins when the χ2 statistic is greater than 27.88, 26.12 and 29.59, respectively, with α = 0.001. * Conservation Factor of each protein. (DOC) [file pone.0043674.s002.doc]

**Table S2. Protein-size distribution of CD8 T cell epitopes, excluding those restricted by A*0201, in HCV, HIV and IAV**

| **HCV** |  |  |  |  |  |
| --- | --- | --- | --- | --- | --- |
| **Protein** | **Protein length** | ***CF**** | **Observed epitopes** | **Expected epitopes** | **χ^2^** |
| Core | 191 | 0,95 | 18 | 7.33 | 15.53 |
| E1 | 192 | 0,58 | 6 | 7.41 | 0.27 |
| E2 | 364 | 0,71 | 16 | 14.30 | 0.20 |
| p7 | 64 | 0,68 | 4 | 2.21 | 1.44 |
| NS2 | 218 | 0,59 | 12 | 8.42 | 1.52 |
| NS3 | 632 | 0,89 | 30 | 25.09 | 0.96 |
| NS4a | 55 | 0,83 | 2 | 1.85 | 0.01 |
| NS4b | 262 | 0,84 | 8 | 10.19 | 0.47 |
| NS5a | 449 | 0,75 | 9 | 17.72 | 4.29 |
| NS5b | 592 | 0,81 | 13 | 23.48 | 4.68 |
| Total | 3019 |  | 118 | 118 | 29.37 |
| **HIV** |  |  |  |  |  |
| **Protein** | **Protein length** | ***CF**** | **Observed epitopes** | **Expected epitopes** | **χ^2^** |
| Gag | 500 | 0,68 | 71 | 33.58 | 41.69 |
| Pol | 1001 | 0,84 | 55 | 67.78 | 2.41 |
| Vif | 192 | 0,75 | 4 | 12.56 | 5.83 |
| Vpr | 96 | 0,74 | 4 | 6.01 | 0.67 |
| Tat | 86 | 0,63 | 4 | 5.26 | 0.30 |
| Rev | 116 | 0,57 | 4 | 7.37 | 1.54 |
| Vpu | 82 | 0,45 | 1 | 5.05 | 3.25 |
| Env | 856 | 0,54 | 42 | 57.88 | 4.36 |
| Nef | 206 | 0,62 | 24 | 13.51 | 8.13 |
| Total | 3135 |  | 209 | 209 | 68.19 |
| **IAV** |  |  |  |  |  |
| **Protein** | **Protein length** | ***CF**** | **Observed epitopes** | **Expected epitopes** | **χ^2^** |
| PB2 | 759 | 0,98 | 6 | 11.48 | 2.61 |
| PB1 | 757 | 0,1 | 11 | 11.45 | 0.02 |
| PB1F2 | 87 | 0,84 | 1 | 1.21 | 0.04 |
| PA | 716 | 0,98 | 5 | 10.82 | 3.13 |
| HA | 566 | 0,88 | 8 | 8.53 | 0.03 |
| NP | 498 | 0,99 | 16 | 7.49 | 9.67 |
| NA | 452 | 0,92 | 4 | 6.81 | 1.16 |
| M1 | 252 | 0,99 | 10 | 3.73 | 10.54 |
| M2 | 97 | 0,89 | 2 | 1.36 | 0.30 |
| NS1 | 230 | 0,83 | 5 | 3.39 | 0.76 |
| NS2 | 121 | 0,92 | 0 | 1.23 | 1.73 |
| Total | 4537 | 10,22 | 68 | 68 | 30.00 |
